# Gastric Cancer Surgery Before and During the COVID-19 Pandemic in Turkey: A Multicenter Comparison of Prognostic Factors, Mortality, and Survival

**DOI:** 10.3390/medicina61081336

**Published:** 2025-07-24

**Authors:** Yasin Dalda, Sami Akbulut, Zeki Ogut, Serkan Yilmaz, Emrah Sahin, Ozlem Dalda, Adem Tuncer, Zeynep Kucukakcali

**Affiliations:** 1 Department of Surgery, Liver Transplant Institute, Inonu University Faculty of Medicine, 44280 Malatya, Türkiye; 2 Department of Biostatistics and Medical Informatics, Inonu University Faculty of Medicine, 44280 Malatya, Türkiye; 3 Department of Surgery, Elazığ Fethi Sekin City Hospital, 23300 Elazig, Türkiye; 4 Department of Surgery, Firat University Faculty of Medicine, 23119 Elazig, Türkiye; 5 Department of Pathology, Inonu University Faculty of Medicine, 44280 Malatya, Türkiye

**Keywords:** gastric cancer, COVID-19 pandemic, prognostic factors, tumor staging, mortality, survival analysis

## Abstract

*Background/Objectives:* The COVID-19 pandemic disrupted global cancer care. This study compared gastric cancer surgical outcomes before and during the pandemic in Turkey. We also aimed to analyze the impact of the pandemic and factors on survival and mortality in gastric cancer patients. *Materials and Methods:* This retrospective, multicenter cohort study included 324 patients from three tertiary centers in Turkey who underwent gastric cancer surgery between January 2018 and December 2022. Patients were stratified into Pre-COVID-19 (*n* = 150) and COVID-19 Era (*n* = 174) groups. Comprehensive demographic, surgical, pathological, and survival data were analyzed. To identify factors independently associated with postoperative mortality, a multivariable logistic regression model was applied. For evaluating predictors of long-term survival, multivariable Cox proportional hazards regression analysis was conducted. *Results:* The median time from diagnosis to surgery was comparable between groups, while the time from surgery to pathology report was significantly prolonged during the pandemic (*p* = 0.012). Laparoscopic surgery (*p* = 0.040) and near-total gastrectomy (*p* = 0.025) were more frequently performed in the Pre-COVID-19 group. Although survival rates between groups were similar (*p* = 0.964), follow-up duration was significantly shorter in the COVID-19 Era (*p* < 0.001). Comparison between survivor and non-survivor groups showed that several variables were significantly associated with mortality, including larger tumor size (*p* < 0.001), greater number of metastatic lymph nodes (*p* < 0.001), elevated preoperative CEA (*p* = 0.001), CA 19-9 (*p* < 0.001), poor tumor differentiation (*p* = 0.002), signet ring cell histology (*p* = 0.003), lymphovascular invasion (*p* < 0.001), and perineural invasion (*p* < 0.001). Multivariable logistic regression identified total gastrectomy (OR: 2.14), T4 tumor stage (OR: 2.93), N3 nodal status (OR: 2.87), and lymphovascular invasion (OR: 2.87) as independent predictors of postoperative mortality. Cox regression analysis revealed that combined tumor location (HR: 1.73), total gastrectomy (HR: 1.56), lymphovascular invasion (HR: 2.63), T4 tumor stage (HR: 1.93), N3 nodal status (HR: 1.71), and distant metastasis (HR: 1.74) were independently associated with decreased overall survival. *Conclusions:* Although gastric cancer surgery continued during the COVID-19 pandemic, some delays in pathology reporting were observed; however, these did not significantly affect the timing of adjuvant therapy or patient outcomes. Importantly, pandemic timing was not identified as an independent risk factor for mortality in multivariable logistic regression analysis, nor for survival in multivariable Cox regression analysis. Instead, tumor burden and aggressiveness—specifically advanced stage, lymphovascular invasion, and total gastrectomy—remained the primary independent determinants of poor prognosis. While pandemic-related workflow delays occurred, institutional adaptability preserved oncologic outcomes.

## 1. Introduction

Coronavirus disease 2019 (COVID-19), caused by SARS-CoV-2, emerged in late 2019 and rapidly became a global pandemic. The World Health Organization declared it a pandemic on 11 March 2020, as health systems worldwide faced severe disruptions [1,2,3,4,5,6,7]. With the progression of the pandemic, healthcare infrastructures worldwide experienced critical strain [8]. Alongside public health interventions such as lockdowns, international travel restrictions, physical distancing, and widespread use of face masks, hospitals adopted radical internal precautions [9,10,11]. Most institutions redirected clinical capacity and human resources to manage COVID-19, leading to a widespread reallocation of health system priorities. As a consequence, the diagnosis and management of non-COVID-19 conditions—including chronic diseases and malignancies—suffered substantial setbacks [12,13,14,15]. Cancer care was particularly vulnerable to these disruptions [16]. Delays in diagnostic workups, treatment initiation, and surgical procedures generated concerns about compromised clinical outcomes and increased cancer-related mortality [17]. These delays affected multiple cancer types, including breast, colorectal, lung, esophageal, and gastric malignancies. Gastric cancer, which constitutes the focus of this study, is of particular concern due to its often asymptomatic course and late-stage presentation [18,19,20,21,22,23,24,25]. In an effort to mitigate the potential adverse outcomes of healthcare interruptions, various international and national oncology societies issued consensus guidelines to preserve essential cancer services during the pandemic.

Gastric cancer continues to be a major global health burden, ranking fifth both in cancer incidence and cancer-related deaths. According to the GLOBOCAN 2022 statistics published by the International Agency for Research on Cancer, gastric cancer accounted for approximately 659,853 deaths worldwide in that year alone [26]. Its risk factors include Helicobacter pylori infection, a positive family history, chronic atrophic gastritis, intestinal metaplasia, tobacco and alcohol use, and prior history of gastric ulcers. In high-risk populations, upper gastrointestinal endoscopy plays a crucial role in early diagnosis and significantly improves survival. Screening programs targeting such individuals can reduce mortality by 30% to 67%, primarily by identifying lesions at curable stages [27,28,29,30].

The COVID-19 pandemic, which severely disrupted healthcare services globally, also significantly affected the screening, diagnosis, and treatment of patients with gastric cancer. In many countries, elective and even some oncologic surgeries were postponed or canceled, both to reallocate resources toward COVID-19 care and to reduce viral transmission risk [31,32]. It is estimated that approximately 38% of oncologic surgeries were delayed or canceled during this period [33]. As with many other malignancies, surgical intervention remains the cornerstone of curative treatment for gastric cancer. In Turkey, following the first confirmed COVID-19 case, national health authorities implemented similar precautionary strategies, leading to postponement of elective procedures including oncologic surgeries [21,34]. In the context of gastric cancer, healthcare disruptions during the COVID-19 pandemic in Turkey led to significant reductions in endoscopy procedures due to concerns over viral transmission, contributing to delayed diagnoses and stage migration. Operating room capacity was constrained by the redeployment of surgical teams and intensive care resources to COVID-19 care, leading to postponed surgeries and interruptions in multidisciplinary cancer management. These specific disruptions highlight the vulnerability of gastric cancer patients during the pandemic.

Although early-phase studies have documented the short-term effects of the pandemic on cancer care, evidence regarding long-term survival outcomes in gastric cancer remains limited [16,32,35,36]. Studies from various countries have reported significant reductions in early-stage diagnoses and increased rates of advanced-stage presentations, yet few have directly linked surgical delays to survival outcomes in gastric cancer patients. While some meta-analyses suggest that delays up to eight weeks may not significantly affect prognosis, especially in early-stage cases, the impact on more advanced disease is uncertain. This persistent knowledge gap challenges clinicians in making evidence-based decisions about safe delays in surgery or treatment, resource allocation, and patient counseling during health crises like the COVID-19 pandemic. Therefore, the primary aim of this multicenter study is to compare gastric cancer patients operated during the Pre-COVID-19 and COVID-19 Era periods in terms of demographic, clinical, and histopathological characteristics. As a secondary objective, the study also investigates factors associated with postoperative mortality and risk factors influencing survival in gastric cancer patients, incorporating the pandemic period as an independent variable in the analyses. Based on this primary objective, we hypothesized that pandemic-related delays would worsen survival, independent of tumor characteristics.

## 2. Materials and Methods

### 2.1. Type, Duration, and Location of the Study

This descriptive and retrospective study includes patients who underwent surgical treatment for gastric cancer between 2 January 2018 and 19 December 2022. This multicenter study was conducted across three institutions: Inonu University, Fırat University, and Fethi Sekin City Hospital, Turkey. All surgeries were performed in the Departments of General Surgery, providing a heterogeneous patient population and allowing for comprehensive clinical data collection.

### 2.2. Determination of the Research Universe and Study Group

Based on data obtained from the hospital information and management systems of the three participating centers in this retrospective study, a total of 324 patients who underwent surgical treatment for gastric cancer between 2 January 2018 and 19 December 2022 were included. Of these, 150 patients who received surgery between 2 January 2018 and 5 March 2020 were classified as the Pre-COVID-19 group. The remaining 174 patients, who underwent gastric cancer surgery between 15 March 2020 and 19 December 2022, were categorized as the COVID-19 Era group.

### 2.3. Inclusion and Exclusion Criteria

This study included patients diagnosed with gastric cancer who underwent surgical treatment at the three aforementioned centers and whose postoperative follow-up and oncological management were conducted at the same institutions. Exclusion criteria were as follows: patients who were diagnosed with gastric cancer but did not undergo surgery; patients who underwent surgery at external institutions and were referred only for oncological treatment; individuals referred solely for histopathological evaluation to the pathology departments of the study centers; and patients with incomplete or insufficient data for statistical analysis.

### 2.4. Definitions, Parameters, and Variables Used in the Study

Patient data—including demographic and clinical characteristics, endoscopic findings, neoadjuvant and adjuvant treatment status, surgical details, tumor histopathology, and postoperative outcomes such as metastasis and mortality—were retrospectively collected from hospital information systems and compiled into a structured Excel database. The time from biopsy-confirmed diagnosis to surgery was defined as the “diagnosis-to-surgery interval,” while the time from surgery to the issuance of the final pathology report was recorded as the “surgery-to-pathology interval.” [21] Follow-up was defined as the period from the date of surgery to the last outpatient clinic visit or telephone contact for surviving patients, or to the date of death for non-surviving patients [21].

Tumor localization was categorized into three anatomical regions based on the dominant site of involvement: proximal, distal, and combined. Proximal tumors were defined as cancers with the center located in the cardia or fundus, while distal tumors were defined as lesions situated in the body, antrum, or pylorus [37]. Tumors involving both proximal and distal regions, or presenting with multiple synchronous foci, were classified as combined gastric tumor. This classification was based on preoperative endoscopic findings, intraoperative evaluations, and final pathological reports.

Neoadjuvant treatment was administered to patients with stage II–III gastric cancer, under 85 years of age, with an ECOG performance status of 0–1 and preserved renal, hepatic, and hematologic function [38]. Surgical interventions were performed using open or laparoscopic techniques depending on the experience of the surgical team and availability of equipment. Regardless of the surgical approach, the extent of gastrectomy was categorized based on tumor location and resection margins as total, near-total, distal subtotal, proximal subtotal, or wedge resection. Staging was conducted according to the 8th edition of the TNM classification system published by the Union for International Cancer Control (UICC)/American Joint Committee on Cancer (AJCC) [39], integrating both clinical and pathological findings. In patients receiving neoadjuvant therapy, T and N staging was based on clinical and radiological evaluation, while in those undergoing upfront surgery, pathological assessment was used. Tumor histologic subtype, presence of mucinous or signet-ring cell components, tumor differentiation grade, total and positive lymph node counts, and the presence of lymphovascular and perineural invasion were documented.

The following variables were examined for comparative analysis: age (years), sex (female, male), comorbidities (e.g., hypertension, diabetes mellitus), American Society of Anesthesiologists (ASA) score, time from diagnosis to surgery (days), time from surgery to pathology report (days), preoperative carcinoembryonic antigen (CEA) and carbohydrate antigen 19-9 (CA 19-9) levels, neoadjuvant chemotherapy, adjuvant chemotherapy, tumor location (proximal, distal, combined), surgical approach (laparoscopic or open), type of gastrectomy (total, near-total, proximal subtotal, distal subtotal, wedge), histological subtype of gastric cancer (adenocarcinoma, poorly cohesive carcinoma, mucinous carcinoma, signet-ring cell carcinoma, neuroendocrine tumor), tumor differentiation (well, moderate, poor), tumor size (cm), total lymph nodes, metastatic lymph nodes, presence of lymphovascular invasion and perineural invasion, TNM stage, length of hospital stay (days), postoperative complications, follow-up (days), and outcome (surviving or non-surviving). 

### 2.5. Study Protocol and Ethics Committee Approval

This study involving human participants was conducted in accordance with the ethical standards of both institutional and national research ethics committees, as well as the 1964 Declaration of Helsinki and its subsequent revisions or comparable ethical guidelines. This study was designed as a retrospective case-control study; therefore, according to ethical regulations, obtaining ethics committee approval and individual patient consent is not required. The Strengthening the Reporting of Observational Studies in Epidemiology, or STROBE, guidelines were followed to identify potential sources of bias and to ensure the overall methodological quality of the study [40].

### 2.6. Statistical Analysis

All statistical analyses were performed using IBM SPSS Statistics version 26.0, IBM Corporation, Armonk, NY, USA. The normality of distribution for continuous variables was assessed using the Shapiro–Wilk test. Non-normally distributed continuous variables were summarized as medians with 95% confidence intervals. Categorical variables were summarized as frequencies and percentages. Since parametric assumptions were not met for continuous variables, non-parametric methods were employed, and group comparisons were performed using the Mann–Whitney U test. Categorical variables were compared using either the Chi-square test or Fisher’s exact test, as appropriate. Survival analysis was conducted using the Kaplan–Meier method (months used for estimate), and differences between groups were evaluated with the log-rank test. To identify independent predictors of mortality, multivariable logistic regression analysis was performed, whereas independent prognostic factors affecting survival time were assessed using multivariable Cox proportional hazards regression analysis. Variables with a *p*-value < 0.05 in univariate analysis were included in both the multivariable logistic regression and the multivariable Cox proportional hazards regression models, using a backward elimination approach. Model calibration for logistic regression was assessed with the Hosmer–Lemeshow test, and results were reported as odds ratios (ORs) with 95% confidence intervals (CIs). Hazard ratios (HRs) with 95% confidence intervals were reported for the Cox model. A *p*-value < 0.05 was considered statistically significant.

## 3. Results

### 3.1. Demographic and Clinicopathological Characteristics of the Entire Cohort

In this study, data from a total of 324 patients who underwent surgery for gastric cancer were retrospectively analyzed. The median age of the cohort was 63 years (95% CI: 62–66), and 63.9% were male. The median time from diagnosis to surgery was 16 days (95% CI: 15–19), and the median time from surgery to the issuance of the final pathology report was 24 days (95% CI: 22–28). The median tumor size was 52 mm (95% CI: 50–56). The median follow-up duration was 1132 days (95% CI: 960–1210), and the median length of hospital stay was 9 days (95% CI: 9–10). A median of 29 (95% CI: 28–32) lymph nodes were harvested, with a median of 4 (95% CI: 3–6) positive regional lymph nodes.

Regarding preoperative tumor markers, the median carcinoembryonic antigen (CEA) level was 1.7 ng/mL (95% CI: 1.53–1.9), and the median carbohydrate antigen 19-9 (CA 19-9) level was 11.13 U/mL (95% CI: 9.2–13.6). Histopathological examination revealed poorly differentiated tumors in 53.1% of patients, signet-ring cell carcinoma components in 24.7%, perineural invasion in 66.4%, and lymphovascular invasion in 79.9%. The most frequent T stage was T4 (41.0%), and the most common N stage was N3 (42.8%). Distant metastasis (M1) was present in 8.6% of cases. According to TNM staging, stage IIIB (22.0%) and IIIC (16.0%) were the most prevalent.

Laparoscopic surgery was performed in 42.6% of patients, and the most common type of resection was total gastrectomy (47.8%). Neoadjuvant therapy was administered to 21.0% of patients, while 67.5% received adjuvant therapy. Postoperative complications occurred in 19.1% of the cohort. The descriptive statistics for these variables are summarized in Table 1 and Table 2.

### 3.2. Comparison Between Pre-COVID-19 and COVID-19 Era Patient Groups

Patients were divided into two groups: Pre-COVID-19 (*n* = 150) and COVID-19 Era (*n* = 174). Comparison between the groups revealed several notable differences. There were no statistically significant differences in age, sex, comorbidities, tumor location, or histopathological features. Although the time from diagnosis to surgery was comparable between groups (*p* = 0.151), the time from surgery to pathology report was significantly longer during the COVID-19 Era (22 days vs. 27 days; *p* = 0.012). The median follow-up duration was significantly shorter in the COVID-19 Era group (1403 days vs. 994.5 days; *p* < 0.001).

In terms of surgical technique, laparoscopic surgery was more commonly performed in the Pre-COVID-19 group (48.7% vs. 37.4%; *p* = 0.040). Additionally, the rate of near-total gastrectomy was significantly higher in the Pre-COVID-19 group (22.7% vs. 10.3%; *p* = 0.025). No significant differences were found between the groups in terms of pathological staging, perineural invasion, lymphovascular invasion, tumor differentiation grade, or the presence of signet-ring cell components. Although the rate of distant metastasis was higher in the COVID-19 Era group, the difference did not reach statistical significance (6.0% vs. 11.1%; *p* = 0.160). The overall TNM staging distribution was also comparable between groups.

These findings suggest that the COVID-19 pandemic had a limited impact on timely access to surgery but led to significant delays in pathology report completion and a notable reduction in follow-up duration. Moreover, the choice of surgical technique, particularly the frequency of laparoscopic surgery and near-total gastrectomy, appears to have shifted during the pandemic period. The comparative results between the Pre-COVID-19 and COVID-19 Era groups are presented in Table 3 and Table 4.

### 3.3. Comparison of Survivors and Non-Survivors with Gastric Cancer

A comparison between surviving (*n* = 143) and non-surviving (*n* = 181) patients revealed several key prognostic factors associated with survival. Tumor size was significantly smaller in survivors (42 mm vs. 58.5 mm, *p* < 0.001), and the number of metastatic lymph nodes was notably lower (1 vs. 9, *p* < 0.001). Preoperative levels of CEA (1.5 vs. 2.0; *p* = 0.001) and CA 19-9 (8.2 vs. 15.87; *p* < 0.001) were significantly higher in non-survivors.

Advanced tumor stages were more prevalent in the non-surviving group, particularly T4 (57.4% vs. 20.1%; *p* < 0.001), N3 (60.2% vs. 20.0%; *p* < 0.001), and M1 (12.8% vs. 3.5%; *p* = 0.003) stages. Histopathological features, including the presence of signet ring cell carcinoma (28.7% vs. 14.7%; *p* = 0.003), poor differentiation (60.3% vs. 42.9%; *p* = 0.002), perineural invasion (80.7% vs. 47.3%; *p* < 0.001), and lymphovascular invasion (93.2% vs. 62.1%; *p* < 0.001), were also significantly more common among non-survivors.

Regarding tumor location, combined anatomical distribution was more prevalent among non-survivors (11.0% vs. 4.2%, *p* = 0.046). Laparoscopic surgery was more frequently performed in the surviving group (50.4% vs. 36.5%; *p* = 0.012). Notably, wedge resection was observed only among survivors. No statistically significant difference was found between groups in terms of postoperative complications.

Lower ASA scores (ASA I) were more frequent among survivors, whereas higher ASA scores (ASA IV) were more prevalent among non-survivors (*p* = 0.017). TNM staging showed a strong correlation with survival: patients with early-stage disease had higher survival rates, while those with stage IIIB, IIIC, and IV disease experienced markedly increased mortality. In addition, median follow-up duration was significantly longer in the surviving group (1785 vs. 494 days; *p* < 0.001). These findings indicate that the most critical determinants of survival in gastric cancer patients are tumor biological behavior, extent of systemic spread, and the ability to perform radical surgical resection (e.g., total or near-total gastrectomy). The comparison between surviving and non-surviving patients is detailed in Table 5 and Table 6.

### 3.4. Survival Trends: Kaplan–Meier Estimates

**Figure 1:**
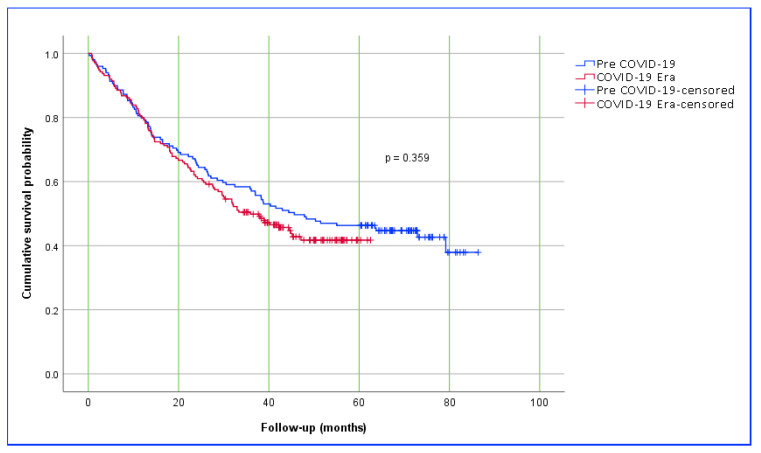
Kaplan–Meier estimate of overall survival for Pre-COVID-19 and COVID-19 Era groups, with axes labeled as “overall survival probability” (*Y*-axis) and “Follow-up time (months)” (*X*-axis).

When median survival durations were analyzed, patients who underwent surgery in the Pre-COVID-19 period had a median survival of 45.6 (25.9–65.4) months, whereas this duration was 35.9 (25.5–46.3) months for those operated on during the COVID-19 Era. Although the difference between the two groups was not statistically significant (*p* = 0.359), the 10 months decrease in median survival is notable and may reflect the indirect consequences of the COVID-19 pandemic. This trend was also supported by the Kaplan–Meier survival analysis. Examination of the survival curves revealed that patients in the Pre-COVID-19 group tended to have longer survival compared to those in the COVID-19 Era group, although this difference did not reach statistical significance. The divergence between the survival curves became more apparent after the second year. This observation suggests that diagnostic delays, restricted access to surgical interventions, and disruptions in healthcare services during the pandemic may have had a negative long-term impact on survival outcomes. Kaplan–Meier survival curves and group-specific survival times are summarized in Table 7 and Figure 1.

### 3.5. Regression Analysis of Factors Affecting Mortality and Survival

To identify independent variables associated with mortality, a multivariable logistic regression analysis was performed. This analysis revealed several significant predictors among patients with gastric cancer (Table 8). Patients who underwent total gastrectomy had significantly higher odds of mortality (OR: 2.14; 95% CI: 1.21–3.77; *p* = 0.009). The presence of T4 stage tumors (OR: 2.93; 95% CI: 1.58–5.45; *p* = 0.001) and N3 nodal involvement (OR: 2.87; 95% CI: 1.53–5.35; *p* = 0.001) were also strongly associated with increased odds of mortality. In addition, lymphovascular invasion emerged as an independent variable significantly associated with higher odds of mortality (OR: 2.87; 95% CI: 1.29–6.40; *p* = 0.010). Model calibration was evaluated using the Hosmer–Lemeshow test (Chi^2^ = 3.332; *p* = 0.912).

To determine prognostic factors influencing overall survival, a multivariable Cox proportional hazards regression analysis was conducted (Table 9). The analysis identified several independent prognostic factors associated with decreased survival: combined tumor location (HR: 1.73; 95% CI: 1.01–2.96; *p* = 0.046), total gastrectomy (HR: 1.56; 95% CI: 1.11–2.18; *p* = 0.010), lymphovascular invasion (HR: 2.63; 95% CI: 1.36–5.06; *p* = 0.004), T4 tumor stage (HR: 1.93; 95% CI: 1.34–2.80; *p* < 0.001), N3 nodal involvement (HR: 1.71; 95% CI: 1.17–2.48; *p* = 0.005), and the presence of distant metastasis at diagnosis (HR: 1.74; 95% CI: 1.07–2.83; *p* = 0.025).

These findings highlight the prognostic significance of lymphovascular invasion, advanced primary tumor size (T4), extensive regional lymph nodes involvement (N3), and distant metastasis at diagnosis, all of which were independently associated with increased odds of mortality and reduced overall survival. Furthermore, surgical characteristics and anatomical distribution—especially tumors involving both proximal and distal regions—were also linked to poorer long-term outcomes.

## 4. Discussion

The COVID-19 pandemic has affected millions of people worldwide, delivering significant blows to the healthcare systems of many countries. Due to the limited knowledge about the virus, its high transmissibility, and elevated mortality rates, particularly among patients with comorbidities, both financial and human resources in healthcare were largely redirected to the management of COVID-19 patients, resulting in the neglect of other medical conditions [41,42]. The restrictions implemented by many governments, the recommendations to visit hospitals only for emergencies, and the widespread fear of infection in the community led to delays in the diagnosis and treatment of many diseases [43,44]. These delays had the most pronounced impact on oncology patients. There is a growing body of literature indicating that cancer patients were neglected during the pandemic and that elective cancer surgeries, particularly in early-stage cases, were frequently postponed [22]. Moreover, it is known that patients with gastrointestinal system cancers, especially gastric cancer, are at a higher risk of contracting COVID-19 and facing increased mortality compared to non-cancer patients due to their compromised immune systems [45,46,47,48].

Although one of the most significant causes of diagnostic delay was the implementation of lockdown measures and the fear of hospital visits—particularly among patients with chronic conditions—the most critical factor was the substantial reduction in endoscopic procedures used in gastric cancer screening, due to the aerosol transmission risk associated with COVID-19. The Japan Gastroenterological Endoscopy Society recommended postponing non-urgent gastrointestinal endoscopic procedures [49]. Studies comparing the pandemic period with Pre-COVID-19 have reported a 42% to 80% decrease in the number of endoscopies performed [42,50,51,52,53,54,55,56].

Naturally, the decrease in endoscopy rates was paralleled by a decline in the number of newly diagnosed gastric cancer cases, a reduction in early-stage diagnoses, a rise in advanced-stage disease presentations, and an increase in complicated cases requiring more frequent emergency or palliative surgical interventions [57,58,59]. In fact, a multinational study reported by Herrera-Kok et al. [59], involving 145 centers from 50 countries, confirmed these global disruptions by reporting a 43.4% decrease in multidisciplinary team meetings, a 54.5% drop in elective gastrectomies, and increases in clinical stage migration (29.0%), metastatic disease rates (33.8%), the need for definitive palliative treatment (26.9%), urgent surgeries (26.9%), and palliative surgeries (16.6%) [59]. These results highlight the widespread and multifactorial impact of the COVID-19 pandemic on the surgical management and outcomes of gastric cancer patients worldwide. Since the current study focused on the management of patients already diagnosed with gastric cancer, it did not address delays in diagnosis or the decrease in the number of diagnosed patients. Therefore, only general trends from the literature regarding these aspects were presented. In this context, unlike many studies in the literature, the findings of the present study suggest that the pandemic did not significantly impact cancer biology-related parameters (such as tumor stage, pathological grade, or extent of local invasion). However, definitive evidence will require long-term, multicenter studies and systematic analyses.

Several countries reported a decrease in the annual number of surgeries during the pandemic, accompanied by a progression in ASA scores and clinical stages among operated patients [60]. However, some studies have shown no significant changes in ASA scores or clinical stage distributions during this period [55,61]. Meanwhile, there has been a noticeable increase in the use of minimally invasive techniques, diagnostic surgeries, and preoperative chemotherapy. These findings collectively highlight the multifaceted and cumulative impact of the pandemic on cancer care delivery, often contributing to a gradual deterioration in patients’ clinical conditions. In the present study, no significant differences were found between the Pre-COVID-19 and COVID-19 Era groups in terms of basic demographic and clinical features, ASA scores, and tumor characteristics, including TNM staging. Moreover, the rate of neoadjuvant therapy increased from 16.7% in the Pre-COVID-19 group to 24.7% in the COVID-19 Era group (*p* = 0.076), indicating a trend approaching statistical significance. Although this increase did not reach conventional significance thresholds, we believe the result is clinically meaningful, as it may reflect evolving treatment strategies during the pandemic aimed at minimizing patient contact, protecting healthcare personnel, and optimizing surgical planning.

Early diagnosis and prompt initiation of treatment are critically important for survival in gastric cancer [62]. The survival rate exceeds 90% for stage I, drops to approximately 45% for stage III, and falls to 9% for stage IV patients [63]. During the pandemic, it has been reported that the time from diagnosis to surgery was prolonged and there were delays in initiating chemotherapy due to COVID-19-related factors [64]. However, the literature presents conflicting findings regarding these time intervals. Some studies have shown that the interval between diagnosis and treatment does not significantly affect prognosis in gastric cancer [65,66,67,68]. Similarly, various studies have demonstrated that surgical delays of 2, 4, 6, or even 8 weeks do not negatively impact prognosis, especially in stage I and II patients [19,43,69,70,71]. Another meta-analysis also suggested that delaying oncological surgery by less than 8 weeks may not adversely affect overall survival in gastric cancer [48].

If left untreated, early-stage gastric cancer has been reported to progress to advanced stages within 34–44 months [72]. In another study, the progression from stage I to stage II occurred within 34 months, whereas progression from stage III to IV was reported to occur in as little as 1.8 months [19]. To date, there is no specific guideline regarding the optimal timing of surgery in gastric cancer [73]. In a multicenter survey involving 36 hospitals in Italy, the number of surgical procedures significantly decreased during the pandemic, and the interval between patient assessment and surgery nearly doubled [74].

Studies conducted during the COVID-19 pandemic have primarily focused on how delays in diagnosis, neoadjuvant therapy, surgery, and the initiation of adjuvant therapy influence patient outcomes. In the present study, although the time from diagnosis to surgery was similar between the groups, the turnaround time for pathology reporting was slightly longer during the COVID-19 Era, which could have potentially led to modest delays in planning adjuvant therapy. However, a review of patient records indicated that the vast majority of pathology reports were finalized within 30 days, generally falling within the usual postoperative recovery timeframe before initiating adjuvant treatment. Moreover, in the present study, no association was observed between the timing of adjuvant therapy and mortality or survival outcomes. Similar findings have been reported in other malignancies, such as biliary cancers, where delays of up to three or four months in initiating adjuvant therapy did not significantly impact overall survival [75]. To our knowledge, no previous studies have specifically compared the interval between surgery and the completion of pathology reports. In our prior publication on breast cancer, we explored this issue and found no significant differences [21]. These observations suggest that moderate delays in postoperative management might not universally translate into worse outcomes, including in gastric cancer. The discrepancy noted in our study may be related to organizational factors at university hospitals, where pathologists are subspecialized by organ, potentially contributing to variations in reporting times.

During the COVID-19 pandemic, numerous studies have reported a significant decline in the number of surgical procedures performed for gastric cancer, although some contrary findings have also been reported in the literature. Feier et al. [22] reported a 44% reduction in such surgeries during the pandemic. Similarly, hospitals across India observed reductions ranging between 17% and 63%, a study from Tokyo reported a 50% decrease, and Italy experienced a 30% decline [41,69,70,76,77,78,79]. However, Sun et al. [15] reported a 21.4% increase in the number of patients undergoing surgery for gastric cancer during the pandemic period. These disruptions likely contributed to delays in diagnosis and treatment, negatively affecting prognosis and increasing the risk of complications. In the present study, only a slight decrease in patient volume was observed between the Pre-COVID-19 and COVID-19 Era periods within the evaluated timeframe. Notably, consistent with the findings of Arneiro et al. [41], no significant differences were identified in tumor characteristics, morbidity, or mortality between the two periods.

The strict public health measures implemented during the pandemic, in addition to causing diagnostic delays, influenced both the prioritization and modality of cancer treatment. During periods of tightened restrictions, surgical treatment options were often suspended, and patients were redirected toward non-surgical modalities such as chemotherapy whenever possible [64]. One major contributing factor was the reallocation of healthcare personnel to pandemic response, which resulted in a reduced number of available operating rooms for elective procedures [80]. In response to these challenges, several clinical guidelines were issued. The Italian Society of Surgical Oncology recommended neoadjuvant chemotherapy for all patients with cT2N0 gastric cancer and advised continuing medical treatment for those who could tolerate it [81]. In another publication, Kang et al. [19] proposed deferring elective surgery for early-stage gastric cancer patients and utilizing neoadjuvant chemotherapy for locally advanced cases, with surgery planned four weeks after the completion of treatment. Moreover, studies on neoadjuvant chemotherapy in gastric surgery have suggested that extending the waiting period beyond six weeks before surgery may improve complete response rates, without adversely impacting prognosis [82,83]. A study from Turkey also reported an increased use of neoadjuvant therapy in gastric cancer patients treated during the post-pandemic period [61]. As previously discussed, although not statistically significant, the present study demonstrated a modest increase in the use of neoadjuvant therapy, which may reflect evolving treatment strategies during the pandemic.

COVID-19 is primarily transmitted through airborne particles and droplets, raising significant concerns in surgical environments. Early in the pandemic, several studies suggested that laparoscopic procedures might lead to aerosol generation due to carbon dioxide insufflation and surgical smoke escaping through trocars, potentially facilitating aerosol transmission of the virus within the operating room [84,85]. As a result, initial guidance from organizations such as the Intercollegiate General Surgery Guidance on COVID-19 and the Society of Gastrointestinal and Endoscopic Surgeons (SAGES) advised caution or avoidance of laparoscopy; however, later updates acknowledged the absence of definitive evidence supporting this risk [86,87]. Techniques like closed vacuum systems with filtration, as described by Li et al. [77], were introduced to reduce the potential for aerosol dispersion and subsequent viral transmission. In the present study, we observed a statistically significant decline in the use of laparoscopic surgery during the pandemic, likely reflecting both early recommendations discouraging laparoscopy and surgeons’ efforts to minimize operative time and exposure risks.

Although there are studies with opposing findings, several publications in the literature have shown an increase in the proportion of stage III and IV gastric cancers during the pandemic. Solaini et al. [64] reported a higher number of T4- and M1-stage patients during the pandemic compared to the same period before. Arneiro et al. [60] also showed a higher incidence of T3/T4 gastric cancer cases during the pandemic. Seker et al. [55] found higher clinical T, pathological T, and clinical N stages in patients diagnosed during the pandemic. In a multicenter retrospective study from Japan, a 32.9% decrease in stage I and an 11.4% increase in stage IV cases were reported [58]. Shigenobu et al. [88] similarly demonstrated a decline in early-stage and an increase in advanced-stage gastric cancer cases. Feier et al. [22] reported an increase in stage III and IV patients compared to the pre-pandemic period. Other studies in the literature also support these findings, showing a decrease in early-stage cancers and a concurrent increase in advanced-stage disease [41,70,71]. Cınkıl et al. [61] reported an increase in stage IIIB patients treated during the pandemic, although overall TNM stage distributions varied. Conversely, Solaini et al. [64] found no significant impact of the pandemic on TNM staging in their large patient series. Similarly, Sun et al. [15] reported that the pandemic had no significant effect on TNM stage distribution. In the present study, no statistically significant differences were found between the Pre-COVID-19 and COVID-19 Era periods in terms of pathological T, pathological N, clinical M, TNM stage, or detailed histopathological tumor characteristics.

There are only a limited number of studies investigating the direct and indirect impact of the COVID-19 pandemic on mortality and long-term survival in patients with gastric cancer, likely due to the recent onset of the pandemic. Ma et al. [48] conducted a meta-analysis including eight studies and a total of 4052 gastric cancer patients. This study demonstrated that delaying surgery for less than 8 weeks does not adversely affect overall survival or disease-free survival. Moreover, postponing surgery by 4, 6, or even 8 weeks was not significantly associated with reduced overall survival.

A study from Portugal reported higher mortality during the early phase of the pandemic, attributing this increase to indirect effects of the pandemic [89]. Another study also found increased mortality during the pandemic, which was associated with increased comorbidity indices, more advanced T staging, and a higher rate of total gastrectomy [22]. A study from Japan reported an increased risk of mortality in gastric cancer patients during the pandemic, with a mean follow-up of 157 days [88]. In contrast, a study from Germany reported no difference in perioperative morbidity and mortality [90], while a Brazilian study found no significant differences in 30- and 90-day mortality rates [60]. A multicenter study from India reported a slight increase in mortality during the pandemic in 6 out of 16 centers [78].

Across these studies, various contributing factors have been emphasized, including delayed hospital admissions, patients waiting until symptoms worsened, prior COVID-19 infection, diagnostic delays, increased tumor burden, poorer clinical and psychological status of patients, and an increase in complications.

Interestingly, a study from Romania reported better survival in gastric cancer patients operated on during the pandemic period; however, the authors could not explain this finding [91]. In our study, no difference in mortality was observed between patients who underwent cancer surgery before and during the pandemic throughout the follow-up period. Similarly, survival analysis showed comparable results. The 1-, 3-, and 5-year survival rates in the pre-pandemic group were 80.7%, 50.7%, and 46.7%, respectively, while these rates in the pandemic group were 79.9%, 49.8%, and 41.7%, respectively. Nevertheless, it is important to note that some of the patients included in this study have not yet completed the full 5-year follow-up period. In summary, due to the naturally shorter follow-up time in patients treated during the pandemic compared to those in the pre-pandemic period, statistically significant results related to follow-up may be misleading. On the other hand, most gastric cancers are diagnosed at an advanced stage and are inherently associated with high mortality and poor survival. Therefore, more definitive conclusions regarding follow-up outcomes will require long-term data.

Numerous studies in the literature have identified key prognostic factors associated with mortality and long-term survival in patients undergoing surgery for gastric cancer. Among the most consistently reported predictors are advanced tumor size (T4), extensive nodal involvement (N2–N3), lymphovascular and perineural invasion, poor tumor differentiation, and the presence of distant metastases. Additionally, factors such as patient age, comorbidities, and ASA score have also been shown to influence surgical outcomes [92,93,94]. Some studies have shown that TNM staging serves as an independent prognostic factor for survival, while biological markers of tumor aggressiveness such as grading, lymphovascular invasion, and perineural invasion were not found to be significant risk factors [95]. In the current study, multivariable analyses were conducted to evaluate the effect of independent variables on mortality and survival in patients who underwent gastric cancer surgery either the Pre-COVID-19 or COVID-19 Era cohorts. In both the logistic and Cox regression models, the period of surgery (Pre-COVID-19 vs. COVID-19 Era) was included as an independent variable but did not demonstrate a statistically significant association with either mortality or overall survival. In the logistic regression model, total gastrectomy (OR: 2.14), advanced tumor stage T4 (OR: 2.93), extensive nodal involvement N3, and the presence of lymphovascular invasion (OR: 2.87) were associated with increased mortality. Similarly, in the Cox proportional hazards model, the variables were identified as independent risk factors significantly associated with decreased survival, including combined tumor location (HR: 1.73), total gastrectomy (HR: 1.56), lymphovascular invasion, T4 stage (HR: 1.93), N3 nodal disease (HR: 1.71), and the presence of distant metastasis (HR: 1.74). These results suggest that classical oncologic prognostic factors were the main determinants of mortality and survival in this cohort, regardless of the timing of surgery relative to the pandemic.

### Limitations

This study has several limitations. First, the retrospective design inherently limits causal inference and may be subject to selection and information biases. Although the multicenter nature of the study enhances its scope, differences in diagnostic protocols, surgical indications, and postoperative management across the participating centers could have introduced variability. Moreover, precise data on key diagnostic intervals—such as the time from symptom onset to endoscopy or from diagnosis to surgery—were not systematically recorded, preventing detailed analysis of diagnostic delays. Another important limitation is the significantly shorter follow-up duration in the COVID-19-era cohort compared to the Pre-COVID-19 group. This disparity limits our ability to evaluate 5- and 10-year survival outcomes and may underestimate the potential late effects of pandemic-related disruptions. Additionally, although pathology report turnaround times were longer during the COVID-19 Era, determining their true impact on survival and mortality will require highly coordinated, multicenter, and large-scale studies to provide reliable evidence. Finally, the unique healthcare structure and pandemic management strategies in Turkey may limit the generalizability of our findings to other countries with different healthcare systems and COVID-19 protocols.

## 5. Conclusions

This multicenter study evaluated the impact of the COVID-19 pandemic on the diagnosis, treatment, and survival of patients with gastric cancer. In line with the existing literature, the pandemic appeared to disrupt screening programs and delay diagnosis while prompting shifts in surgical practice—most notably, a reduction in laparoscopic procedures—which may have influenced treatment strategies. Nevertheless, our analysis revealed no statistically significant differences in tumor stage, biological behavior, or histopathological features between the pre-pandemic and pandemic groups. While neoadjuvant therapy usage showed a slight increase, although not statistically significant, this trend might carry clinical relevance. Additionally, the observed delay in the availability of pathology reports could potentially influence survival by postponing the initiation of adjuvant therapy. To better understand these long-term outcomes, retrospective cohort studies or registry-based investigations with long-term follow-up periods may offer more practical and informative insights.

Moreover, several clinicopathological factors—including advanced tumor stage (T4), extensive nodal involvement (N3), lymphovascular invasion, total gastrectomy, and distant metastasis (M1)—were found to be independently associated with higher odds of mortality and poorer survival outcomes. These findings align with established prognostic indicators and underscore the importance of accurate staging and comprehensive pathological evaluation in optimizing gastric cancer management.

## Figures and Tables

**Table 1:** Descriptive statistics of continuous clinical parameters in patients with gastric cancer.

Parameters [Median (95% CI)]	Results
From diagnosis to surgery (days)	16 (15–19)
From surgery to pathology report (days)	24 (22–28)
Age (years)	63 (62–66)
Preop CEA	1.7 (1.53–1.9)
Preop CA19-9	11.13 (9.2–13.6)
Tumor size (mm)	52 (50–56)
Total LAP (n)	29 (28–32)
Positive LAP (n)	4 (3–6)
Hospital stay (days)	9(9–10)
Follow-Up (days)	1132 (960–1210)

LAP: Lymphadenopathy; CEA: Carcinoembryonic antigen; CA19-9: Carbohydrate antigen 19-9; CI: Confidence interval.

**Table 2:** Distribution of categorical demographic, clinical, and histopathological characteristics in gastric cancer patients.

Parameters	Categories	Number (%)
Groups	Pre-COVID-19	150 (46.3)
	COVID-19 Era	174 (53.7)
Centers	Inonu University	179 (55.2)
	Firat University	99 (30.6)
	Fethi Sekin State Hospital	46 (14.2)
Gender	Male	207 (63.9)
Comorbidity		
DM	Present	46 (14.3)
HT	Present	79 (24.5)
COPD	Present	39 (12.1)
CAD	Present	44 (13.6)
HBV	Present	15 (4.6)
ASA score	I	23 (7.1)
	II	137 (42.3)
	III	157 (48.5)
	IV	7 (2.2)
Tumor location	Distal	238 (73.5)
	Proximal	60 (18.5)
	Combined	26 (8.0)
Neoadjuvant therapy	Received	68 (21.0)
Type of surgery	Laparoscopic surgery	138 (42.6)
Resection type	Near total	52 (16.0)
	Distal subtotal	104 (32.1)
	Total	155 (47.8)
	Proximal subtotal	7 (2.2)
	Wedge resection	6 (1.9)
Mucinous	Yes	21 (7.1)
Signet ring cell	Yes	73 (24.7)
Tumor differentiation	Well	38 (12.5)
	Moderately	105 (34.4)
	Poorly	162 (53.1)
Perineural invasion	Yes	204 (66.4)
Lymphovascular Invasion	Yes	246 (79.9)
Pathology (T)	1	37 (11.7)
	2	34 (10.8)
	3	115 (36.5)
	4	129 (41.0)
Pathology (N)	0	77 (24.8)
	1	55 (17.7)
	2	46 (14.8)
	3	133 (42.8)
Clinic (M)	M0	296 (91.4)
	M1	28 (8.6)
TNM Stage	IA	26 (8.3)
	IB	18 (5.8)
	IIA	40 (12.8)
	IIB	40 (12.8)
	IIIA	42 (13.4)
	IIIB	69 (22.0)
	IIIC	50 (16.0)
	IV	28 (8.9)
Postoperative complications	Present	62 (19.1)
Adjuvant therapy	Received	218 (67.5)
Outcomes	Alive	143 (44.1)
	Dead	181 (55.9)

T: Tumor size; N: Lymph nodes; M: Metastases; COPD: Chronic obstructive pulmonary disease; DM: Diabetes mellitus; HT: Hypertension; HBV: Hepatitis B virus; ASA: American society of anesthesiologists.

**Table 3:** Comparison of continuous clinical variables between Pre-COVID-19 and COVID-19 Era groups.

Parameters [Median (95% CI)]	Pre-COVID-19	COVID-19 Era	*p* *
From diagnosis to surgery (days)	15 (14–18)	16 (15–20)	0.151
From surgery to pathology report (days)	22 (20–27)	27 (22–31)	0.012
Age (years)	63 (61–67)	62.5 (60–65)	0.742
Preop CEA	1.7 (1.27–2.33)	1.7 (1.53–1.98)	0.804
Preop CA19-9	9.7 (7.7–13.9)	11.8 (9.2–15.4)	0.284
Tumor size (mm)	52 (45–60)	52 (48–60)	0.195
Total LAP (n)	28 (26–32)	29 (28–34)	0.150
Positive LAP (n)	4 (3–7)	4 (2–7)	0.595
Hospital stay (days)	9 (8–10)	9 (9–10)	0.388
Follow Up (days)	1403 (1083–1856)	994.5 (839–1151)	<0.001

* Mann–Whitney U test was used for comparisons between groups.

**Table 4:** Comparison of categorical variables between Pre-COVID-19 and COVID-19 Era groups.

Parameters (%)	Categories	Pre-COVID-19	COVID-19 Era	*p*
Gender	Male	96 (64.0)	111 (63.8)	0.969 *
Comorbidity				
DM	Yes	24 (16.2)	22 (12.6)	0.451 **
HT	Yes	39 (26.4)	40 (23.0)	0.485 *
COPD	Yes	17 (11.5)	22 (12.6)	0.887 **
CAD	Yes	20 (13.5)	24 (13.8)	0.999 **
HBV	Yes	9 (6.0)	6 (3.5)	0.402 **
ASA	1	13 (8.9)	7 (4.0)	0.118 *
	2	62 (42.47)	74 (42.53)	
	3	70 (48.0)	87 (50.0)	
	4	1 (0.7)	6 (3.45)	
Tumor Location	Distal	113 (75.3)	125 (71.8)	0.111 *
	Proksimal	30 (20.0)	30 (17.2)	
	Combined	7 (4.7)	19 (10.9)	
Neoadjuvant Therapy	Yes	25 (16.7)	43 (24.7)	0.076 *
Type of surgery	Laparoscopic surgery	73 (48.7)	65 (37.4)	0.040 *
Resection type	Near total	34 (22.7)	18 (10.3)	0.025 *
	Distal subtotal	44 (29.3)	60 (34.5)	
	Total	64 (42.7)	91 (52.3)	
	Proximal subtotal	4 (2.7)	3 (1.7)	
	Wedge resection	4 (2.7)	2 (1.2)	
Mucinous	Yes	12 (8.0)	9 (5.2)	0.421 **
Signet ring cell	Yes	31 (20.7)	42 (24.1)	0.456 *
Tumor differentiation	Well	18 (12.8)	20 (12.2)	0.691 *
	Moderately	45 (31.9)	60 (36.6)	
	Poorly	78 (55.3)	84 (51.2)	
Perineural Invasion	Yes	91 (63.6)	113 (68.9)	0.330 *
Lymphovascular Invasion	Yes	116 (80.6)	130 (79.3)	0.779 *
Pathology (T)	1	21 (14.0)	16 (9.7)	0.328 *
	2	18 (12.0)	16 (9.7)	
	3	48 (32.0)	67 (40.6)	
	4	63 (42.0)	66 (40.0)	
Pathology (N)	0	37 (24.7)	40 (24.8)	0.928 *
	1	25 (16.7)	30 (18.6)	
	2	24 (16.0)	22 (13.7)	
	3	64 (42.7)	69 (42.9)	
Clinic (M)	M0	141 (94.0)	153 (89.0)	0.160 **
	M1	9 (6.0)	19 (11.1)	
TNM Stage	IA	14 (9.6)	12 (7.2)	0.214 *
	IB	12 (8.2)	6 (3.6)	
	IIA	15 (10.3)	25 (15.0)	
	IIB	15 (10.3)	25 (15.0)	
	IIIA	21 (14.4)	21 (12.6)	
	IIIB	36 (24.7)	33 (19.8)	
	IIIC	24 (16.4)	26 (15.6)	
	IV	9 (6.2)	19 (11.4)	
Postoperative complications	Present	35 (23.3)	27 (15.5)	0.075 *
Adjuvant Therapy	Received	104 (69.8)	114 (65.5)	0.413 *
Outcomes	Alive	66 (44.0)	77 (44.3)	0.964 *
	Dead	84 (56.0)	97 (55.8)	

* Pearson chi-square. ** Yates’ continuity correction.

**Table 5:** Comparison of continuous variables between alive and dead subgroups.

Parameters [Median (95% CI)]	Survivor	Non-Survivor	*p*
From diagnosis to surgery (days)	16 (14–22)	15 (14–19)	0.822
From surgery to pathology report (days)	21 (20–27)	27 (24–31)	0.008
Age (years)	61 (60–65)	64 (62–68)	0.075
Preop CEA	1.5 (1.2–1.7)	2.0 (1.6–2.5)	0.001
Preop CA19-9	8.2 (6.3–10.5)	15.87 (12–22)	<0.001
Tumor size (mm)	42 (40–50)	58.5 (53–63)	<0.001
Total LAP (n)	29.5 (27–33)	28.5 (27–31)	0.774
Positive LAP (n)	1 (1–2)	9 (8–12)	<0.001
Hospital stay (days)	8 (8–9)	10 (10–12)	0.107
Follow-Up (days)	1785 (1679–1860)	494 (414–613)	<0.001

**Table 6:** Comparison of categorical clinical and histopathological features between alive and dead subgroups.

Parameters (%)	Categories	Survivor	Non-Survivor	*p*
Groups	Pre-COVID-19	66 (46.2)	84 (46.4)	0.964 *
	COVID-19 Era	77 (53.9)	97 (53.6)	
Gender	Male	85 (59.4)	122 (67.4)	0.138
Comorbidity				
DM	Yes	17 (11.9)	29 (16.2)	0.272 *
HT	Yes	36 (25.2)	43 (24.0)	0.881 *
COPD	Yes	16 (11.2)	23 (12.9)	0.778 **
CAD	Yes	16 (11.2)	23 (12.9)	0.196 **
HBV	Yes	6 (4.2)	9 (5.0)	0.940 **
ASA	1	14 (9.8)	6 (3.4)	0.017 *
	2	53 (37.1)	83 (46.9)	
	3	75 (52.45)	82 (46.3)	
	4	1 (0.7)	6 (3.4)	
Tumor Location	Distal	113 (79.0)	125 (69.1)	0.046 **
	Proksimal	24 (16.8)	36 (19.9)	
	Combined	6 (4.2)	20 (11.0)	
Neoadjuvant therapy	Received	25 (17.5)	43 (23.8)	0.168 *
Type of surgery	Laparoscopic surgery	72 (50.4)	66 (36.5)	0.012 *
Resection type	Near total	23 (16.1)	29 (16.0)	0.003 *
	Distal subtotal	56 (39.2)	48 (26.5)	
	Total	55 (38.5)	100 (55.3)	
	Proximal subtotal	3 (2.1)	4 (2.2)	
	Wedge resection	6 (4.2)	0 (0.0)	
Mucinous	Yes	8 (5.6)	13 (7.18)	0.727 **
Signet ring cell	Yes	21 (14.7)	52 (28.7)	0.003 **
Tumor differentiation	Well	24 (19.1)	14b (7.8)	0.002 **
	Moderately	48 (38.1)	57 (31.8)	
	Poorly	54 (42.9)	108 (60.3)	
Perineural invasion	Yes	62 (47.3)	142 (80.7)	<0.001 *
Lymphovascular invasion	Yes	82 (62.1)	164 (93.2)	<0.001 **
Pathology (T)	1	29 (20.9)	8 (4.55)	<0.001 *
	2	28 (20.1)	6 (3.4)	
	3	54 (38.9)	61 (34.7)	
	4	28 (20.1)	101 (57.4)	
Pathology (N)	0	54 (40.0)	23 (13.1)	<0.001 *
	1	37 (27.4)	18 (10.2)	
	2	17 (12.6)	29 (16.5)	
	3	27 (20.0)	106 (60.2)	
Clinic (M)	M0	137 (96.5)	157 (87.2)	0.003 **
	M1	5 (3.5)	23 (12.8)	
TNM stage	IA	19 (14.3)	7 (3.9)	<0.001 *
	IB	18 (13.5)	0 (0.0)	
	IIA	24 (18.1)	16 (8.9)	
	IIB	25 (18.8)	15 (8.3)	
	IIIA	17 (12.8)	25 (13.9)	
	IIIB	18 (13.5)	51 (28.3)	
	IIIC	7 (5.3)	43 (23.9)	
	IV	5 (3.8)	23 (12.8)	
Postoperative complications	Yes	24 (16.8)	38 (21.0)	0.415 **
Adjuvant therapy	Received	94 (65.7)	124 (68.9)	0.548

* Pearson chi-square. ** Yates’ continuity correction.

**Table 7:** Survival time comparison between Pre-COVID-19 and COVID-19 Era patient groups.

Group	Median (Months)				*p*
	Estimate	Std. Error	95% CI		
			Lower Bound	Upper Bound	
Pre-COVID-19	45.6	10.1	25.9	65.4	0.359
COVID-19 Era	35.9	5.3	25.5	46.3	
Overall	39.0	4.4	30.4	47.5	

**Table 8:** Adjusted multivariable logistic regression analysis of factors associated with mortality in patients with gastric cancer.

Variables	B	SE	Wald	*p*	OR	95% CI
Total gastrectomy	0.761	0.289	6.918	0.009	2.14	1.21–3.77
Pathology (T4)	1.080	0.316	11.574	0.001	2.93	1.58–5.45
Pathology (N3)	1.053	0.319	10.929	0.001	2.87	1.53–5.35
Lymphovascular invasion	1.053	0.409	6.621	0.010	2.87	1.29–6.40

Hosmer and Lemeshow (Chi-square 3.332; *p* = 0.912).

**Table 9:** Adjusted multivariable cox regression analysis of prognostic factors for survival in patients with gastric cancer.

Variables	B	SE	Wald	*p*	HR	95% CI
Combined location	0.548	0.275	3.967	0.046	1.73	1.01–2.96
Total gastrectomy	0.442	0.172	6.587	0.010	1.56	1.11–2.18
Lymphovascular invasion	0.965	0.335	8.303	0.004	2.63	1.36–5.06
Pathology (T4)	0.656	0.185	12.607	<0.001	1.93	1.34–2.80
Pathology (N3)	0.534	0.192	7.751	0.005	1.71	1.17–2.48
Clinic (M1)	0.554	0.248	5.008	0.025	1.74	1.07–2.83

## Data Availability

The datasets analyzed during the current study are available from the corresponding author on reasonable request.
